# Supplementary material for: Investigation of the Antiremodeling Effects of Losartan, Mirabegron and Their Combination on the Development of Doxorubicin-Induced Chronic Cardiotoxicity in a Rat Model
Source: Int J Mol Sci. 2022 Feb 16;23(4):2201. doi: 10.3390/ijms23042201 (PMC8877618; doi:10.3390/ijms23042201)
Supplement: Supplementary file 1 [file ijms-23-02201-s001.zip › ijms-1593134-SI.pdf]

International Journal of
Molecular Sciences

MDPI

Investigation of the Antiremodeling Effects of Losartan, Mirabegron and Their Combination on the Development of Doxorubicin-Induced Chronic Cardiotoxicity in a Rat Model

Marah Freiwan [1,2], Mónika G. Kovács [1,2], Zsuzsanna Z. A. Kovács [1,2], Gergő Szűcs [1,2], Hoa Dinh [1,2], Réka Losonczi [1,2], Andrea Siska [3], András Kriston [4,5,6], Ferenc Kovács [4,5,6], Péter Horváth [4,5,6], Imre Földesi [3], Gábor Cserni [7], László Dux [2,8,*], Tamás Csont [1,2,†] and Márta Sárközy [1,2,*,†]

1 MEDICS Research Group, Department of Biochemistry, Albert Szent-Györgyi Medical School, University of Szeged, H-6720 Szeged, Hungary; marah.mf.94@gmail.com (M.F.); kovacs.monika.gabriella@med.u-szeged.hu (M.G.K.); kovacs.zsuzsanna@med.u-szeged.hu (Z.Z.A.K.); szucs.gergo@med.u-szeged.hu (G.S.); dinhhoaqa@gmail.com (H.D.); losonczireka1997@gmail.com (R.L.); csont.tamas@med.u-szeged.hu (T.C.)
2 Interdisciplinary Center of Excellence, University of Szeged, H-6720 Szeged, Hungary
3 Department of Laboratory Medicine, Albert Szent-Györgyi Medical School, University of Szeged, H-6720 Szeged, Hungary; siska.andrea@med.u-szeged.hu (A.S.); foldesi.imre@med.u-szeged.hu (I.F.)
4 Synthetic and Systems Biology Unit, Biological Research Centre, Eötvös Loránd Research Network, H-6726 Szeged, Hungary; kriston.andras@single-cell-technologies.com (A.K.); kovacs.ferenc@single-cell-technologies.com (F.K.); peter.horvath@brc.hu (P.H.);
5 Single-Cell Technologies Ltd., H-6726 Szeged, Hungary
6 Institute for Molecular Medicine Finland (FIMM), University of Helsinki, FIN-00014 Helsinki, Finland
7 Department of Pathology, Albert Szent-Györgyi Medical School, University of Szeged, H-6720 Szeged, Hungary; cserni.gabor@med.u-szeged.hu
8 Muscle Adaptation Group, Department of Biochemistry, Albert Szent-Györgyi Medical School, University of Szeged, H-6720 Szeged, Hungary
* Correspondence: dux.laszlo@med.u-szeged.hu (L.D.); sarkozy.marta@med.u-szeged.hu (M.S.)
† These authors contributed to the work equally.

Supplementary Materials

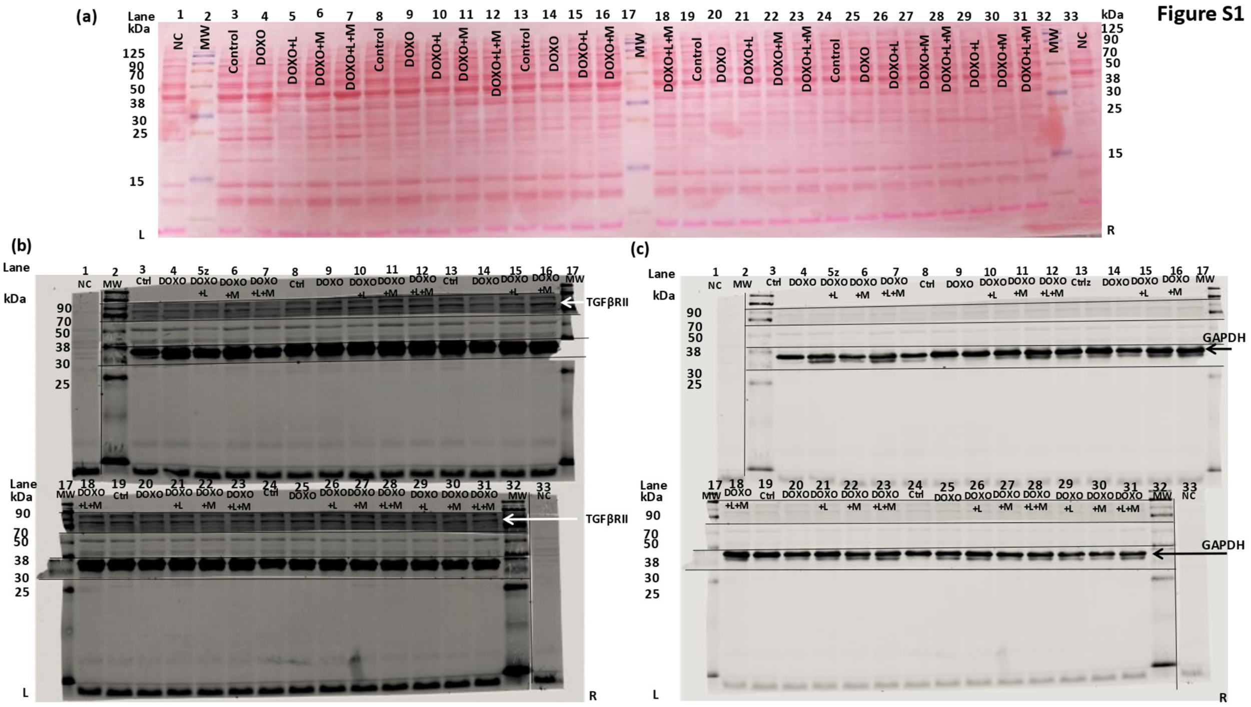

Figure S1. (a) Photo of the uncropped and unmodified Ponceau-stained membrane used later for the detection of transforming factor-beta receptor type II (TGFβRII) and GAPDH. The efficacy of the transfer of proteins onto a nitrocellulose membrane was checked using Ponceau staining. Images

were captured by the camera of an Apple iPhone11. (b) Unmodified Western blot images of transforming factor-beta receptor type II (TGFβRII) and (c) GAPDH. Scanned images were captured with the Odyssey CLx machine and exported with Image Studio 5.2.5 software. Different parts of the same membrane are divided by black lines. The membrane was physically cut in the middle of the molecular weight marker at lane 17 before scanning. Cropped images were used in Figure 5c. Ctrl: control group, DOXO: DOXO only, DOXO+L: losartan-treated DOXO, DOXO+M: mirabegron-treated DOXO, DOXO+L+M: losartan plus mirabegron-treated DOXO, MW: molecular weight marker, NC: negative control, L: left, R: right.

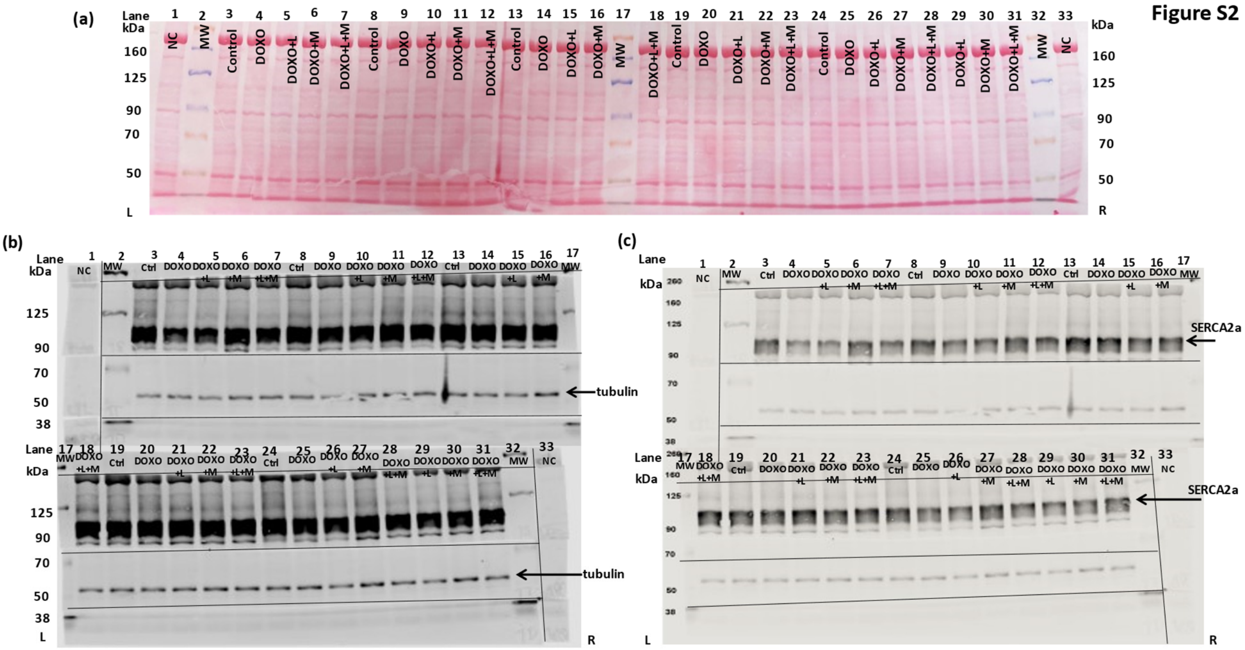

Figure S2. (a) Photo of the uncropped and unmodified Ponceau-stained membrane used later for the detection of sarcoendoplasmic reticulum calcium ATPase 2a isoform (SERCA2a) and tubulin. The efficacy of the transfer of proteins onto a nitrocellulose membrane was checked using Ponceau staining. Images were captured by the camera of an Apple iPhone11. (b) Unmodified Western blot images of tubulin and (c) sarcoendoplasmic reticulum calcium ATPase 2a isoform (SERCA2a). Scanned images were captured with the Odyssey CLx machine and exported with Image Studio 5.2.5 software. Different parts of the same membrane are divided by black lines. The membrane was physically cut in the middle of the molecular weight marker at lane 17 before scanning. Cropped images were used in Figure 6a. Ctrl: control group, DOXO: DOXO only, DOXO+L: losartan-treated DOXO, DOXO+M: mirabegron-treated DOXO, DOXO+L+M: losartan plus mirabegron-treated DOXO, MW: molecular weight marker, NC: negative control, L: left, R: right.

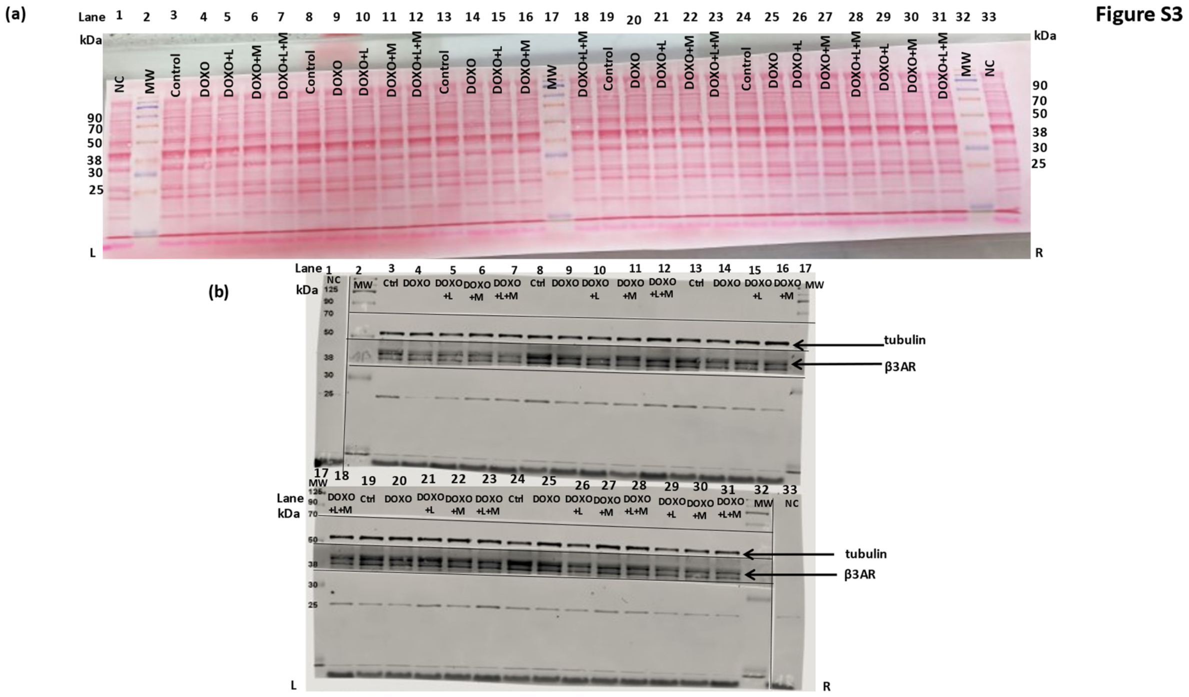

Figure S3. (a) Photo of the uncropped and unmodified Ponceau-stained membrane used later for the detection of beta-3 adrenoreceptor (β3AR) and tubulin. The efficacy of the transfer of proteins onto a nitrocellulose membrane was checked using Ponceau staining. Images were captured by the camera of an Apple iPhone11. (b) Unmodified Western blot images of beta-3 adrenoreceptor (β3AR) and tubulin. Scanned images were captured with the Odyssey CLx machine and exported with Image Studio 5.2.5 software. Different parts of the same membrane are divided by black lines. The membrane was physically cut in the middle of the molecular weight marker at lane 17 before scanning. Cropped images were used in Figure 6b. Ctrl: control group, DOXO: DOXO only, DOXO+L: losartan-treated DOXO, DOXO+M: mirabegron-treated DOXO, DOXO+L+M: losartan plus mirabegron-treated DOXO, MW: molecular weight marker, NC: negative control, L: left, R: right.

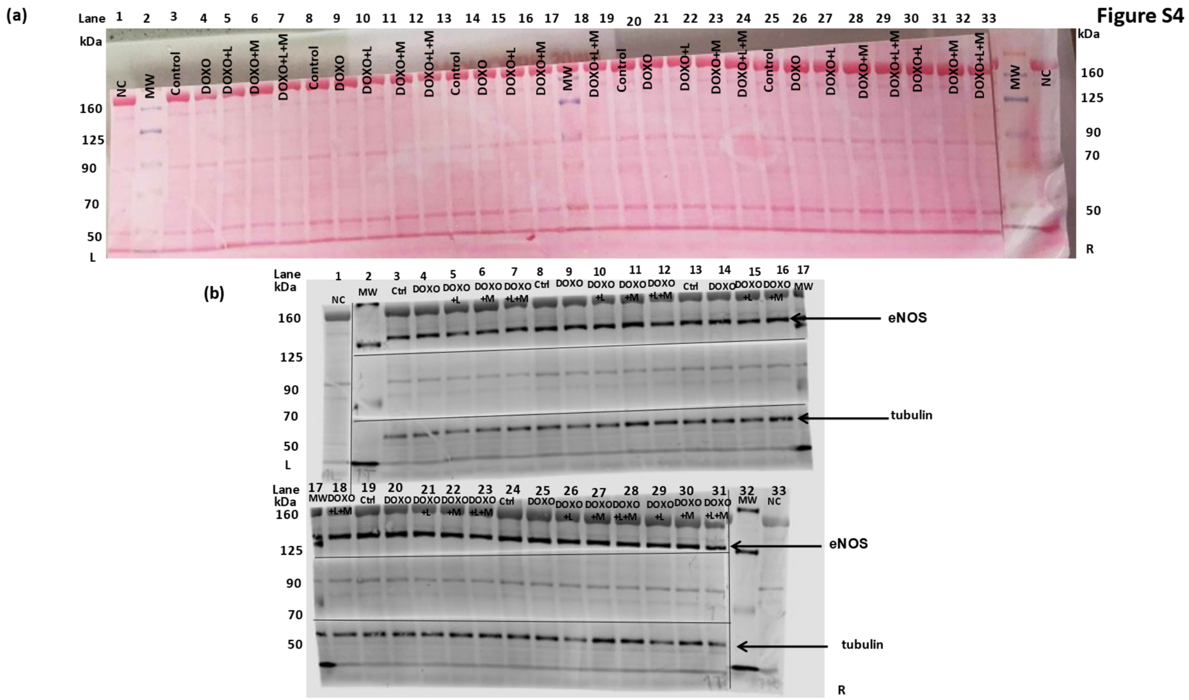

Figure S4. (a) Photo of the uncropped and unmodified Ponceau-stained membrane used later for the detection of endothelial nitric oxide synthase (eNOS) and tubulin. The efficacy of the transfer of

proteins onto a nitrocellulose membrane was checked using Ponceau staining. Images were captured by the camera of an Apple iPhone11. (b) Unmodified Western blot images of endothelial nitric oxide synthase (eNOS) and tubulin. Scanned images were captured with the Odyssey CLx machine and exported with Image Studio 5.2.5 software. Different parts of the same membrane are divided by black lines. The membrane was physically cut in the middle of the molecular weight marker at lane 17 before scanning. Cropped images were used in Figure 6c,e. Ctrl: control group, DOXO: DOXO only, DOXO+L: losartan-treated DOXO, DOXO+M: mirabegron-treated DOXO, DOXO+L+M: losartan plus mirabegron-treated DOXO, MW: molecular weight marker, NC: negative control, L: left, R: right.

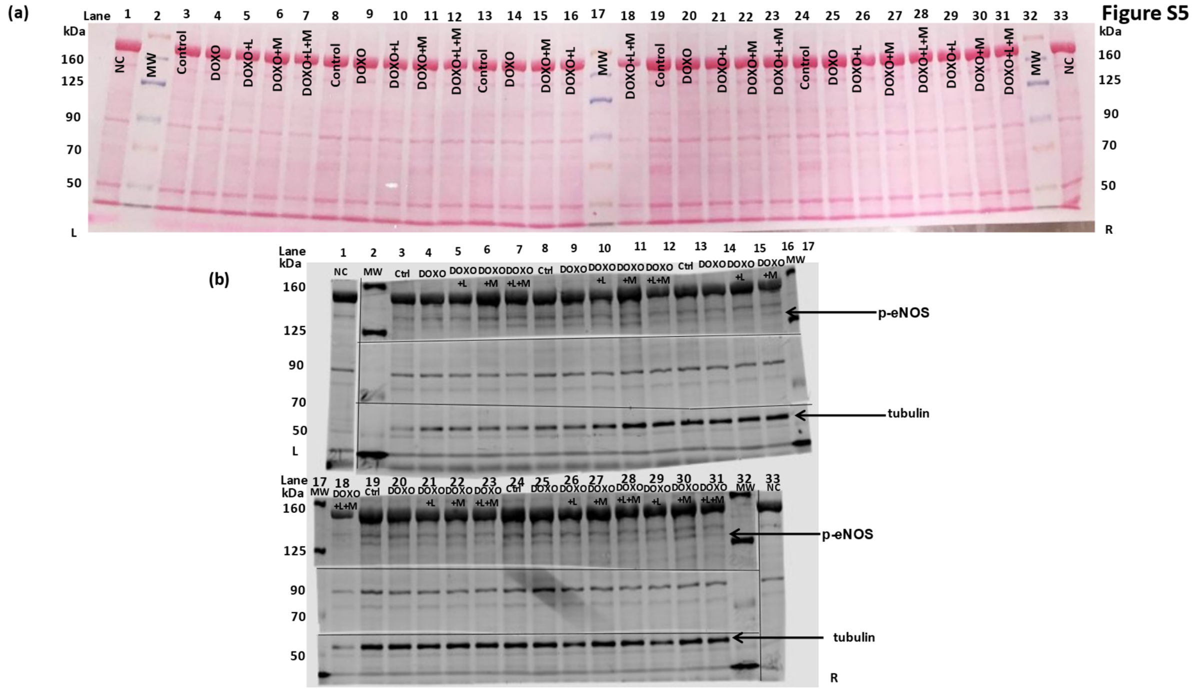

Figure S5. (a) Photo of the uncropped and unmodified Ponceau-stained membrane used later for the detection of phospho-endothelial nitric oxide synthase (p-eNOS) and tubulin. The efficacy of the transfer of proteins onto a nitrocellulose membrane was checked using Ponceau staining. Images were captured by the camera of an Apple iPhone11. (b) Unmodified Western blot images of phospho-endothelial nitric oxide synthase (p-eNOS) and tubulin. Scanned images were captured with the Odyssey CLx machine and exported with Image Studio 5.2.5 software. Different parts of the same membrane are divided by black lines. The membrane was physically cut in the middle of the molecular weight marker at lane 17 before scanning. Cropped images were used in Figure 6d,e. Ctrl: control group, DOXO: DOXO only, DOXO+L: losartan-treated DOXO, DOXO+M: mirabegron-treated DOXO, DOXO+L+M: losartan plus mirabegron-treated DOXO, MW: molecular weight marker, NC: negative control, L: left, R: right.

A detailed description of our Western blot method from Ponceau staining

To avoid any misunderstanding, we would also like to provide a detailed description of the steps following the transfer of the proteins in our Western blot protocol:

1. The efficacy of the transfer of proteins onto a nitrocellulose membrane was checked using Ponceau staining (Figure S1a-S5a).

2. Vertical cuts on the two sides of the membrane were applied to separate the negative controls (NC) to incubate them separately, without the corresponding primary antibody (lanes 1 and 33 on the Ponceau-stained images and the scanned nitrocellulose membranes (Figure S1-S5).

3. We used three lanes (2, 17, and 32 in the Ponceau-stained images and the nitrocellulose membranes) for molecular weight markers (Cameleon Duo Pre-stained Protein Ladder, LI-COR Biosciences, USA) (Figure S1-S5).

4. Horizontal cuts of the membrane were made corresponding to the molecular weight markers. We chose the level of the horizontal cuts precisely to ensure no physical cuts in the region of the molecular weight of the target protein. Please see an example in Figure S3. In the case of the detection of the β3AR, the original membrane was cut into 6 different parts. With 1-1 vertical cut, the negative controls were separated on both sides. Then, 4 horizontal membrane strips were created with 3 horizontal cuts following the line of the 70, 50, and 38 kDa molecular weight markers. The middle of the membrane at lane 17 corresponding to the middle molecular weight marker was cut just before the scanning by the Odyssey CLx machine to half the membrane without cutting any samples. (Please see also Point 11).

5. Then, the membrane strips were blocked in bovine serum albumin (BSA) and were incubated with the specific primary antibodies overnight. Nevertheless, membrane pieces not containing target proteins (e.g., the membrane strips over 70 kDa and under 30 kDa regions in Figure S3) and negative controls were incubated only in BSA overnight.

6. Specific detection of the target protein is well visible on the scans; the fluorescent signal of the target protein is missing at the corresponding kDa region on the negative control lane, which was incubated separately without the specific primary antibody. Please see an example in Figure S3. In the case of the detections of β3AR and tubulin as a loading control, the membrane strip of the 38-50 kDa region was incubated with a primary antibody against β3AR, and the 50-70 kDa region was incubated with a primary antibody against tubulin overnight. The membrane strips over 70 kDa and under 38 kDa regions and the negative controls were incubated in BSA only, without any specific primary antibodies.

7. Antibodies used in our MS are commercially available and well-characterized antibodies TGF-βII receptor II (#79424T, Cell Signaling Technology Inc., USA), SERCA2a (#4388S, Cell Signaling Technology Inc., Danvers, MA, USA), β3AR (AB101095, Abcam PLC, Cambridge, UK), eNOS (#32027S, Cell Signaling Technology Inc., Danvers, MA, USA), p-eNOS (Ser1177, #9570S, Cell Signaling Technology Inc., Danvers, MA, USA) against GAPDH (#2118, Cell Signaling Technology Inc., USA) or α-tubulin (#2144S, Cell Signaling Technology Inc., Danvers, MA, USA).

8. Then, all the membrane pieces were incubated with the secondary antibodies (IRDye 800CW Goat Anti-Rabbit, LI-COR Biosciences, USA).

9. The membrane pieces were united in the original orientation just before the scanning. Then, with a vertical cut, the left and right halves of the membrane were separated in the middle of lane 17, where the middle molecular weight marker was placed (Figure S1-S5). Halving the membrane was necessary to fit the membrane parts into the Odyssey machine for scanning (see also Point 11). All the cuts of the nitrocellulose membrane mentioned above are physical separations of the whole membrane, and the created pieces were united again in the original orientation to scan the membrane as a whole membrane with the Odyssey CLx machine (LI-COR Biosciences, USA).

10. Scanning the membrane pieces together was necessary because the Quantity One 4.4.0 software used for quantitative densitometric evaluation of the fluorescent signals subtracts the background intensity from the band intensity. For the correct quantitative evaluation, each target protein band and the loading control protein should be on the same scan image.

11. The Odyssey CLx machine has an image field size of 25 cm × 25 cm for scanning (https://www.licor.com/bio/odyssey-clx/specifications) (Figure S6), but the electrophoresis gel and membrane (which is suitable for loading our 21 samples) are 30 cm × 7 cm (Figure S6a and S6b). Therefore, to fit the membranes in the Odyssey CLx machine, we cut them in half (with a vertical cut in the middle as mentioned above) just before the scanning (Figure S6a and S6b). No samples were damaged because the molecular weight marker was applied in the middle pocket of the gel in lane 17, and the cut was placed in the middle of the molecular weight marker in lane 17, as also seen on the scanned images (Figure S1-S5). This halved state of the membrane is visible in Figure S6a-g.

Fluorescent signals on the membranes were detected by the Odyssey CLx machine. Therefore, the membrane pieces were placed into the Odyssey CLx machine face down (Figure S6c-f). It means that the surface of the membrane was placed on the glass surface of the scanner, where the color of the molecular weight marker was visible. The right membrane half was placed to the left upper zone of the Odyssey CLx machine, and the left membrane half was placed under the right half of the membrane (Figure S6c-f). Then, the Odyssey CLx machine scanned the two membrane halves together into one digital image. The direction of the scanning was from up to down and from right to left in the Odyssey CLx machine. Due to the aforementioned face down orientation of the membrane halves and the direction of the scanning, the right half of the membrane starts with the halved molecular weight marker lane in the upper position, and the left half of the membrane starts with the left molecular weight marker in the lower position (i.e., image mirrored in the vertical plane compared to the physical membrane). To help understand the positioning, we marked the left (L) and right (R) sides of the Ponceuas-stained membranes, the cut membrane parts, and the scanned digital images of the membranes in the Supplementary Figures. Our scanned images were not modified by digital post-capture mirroring or digital cutting and putting into the original orientation of the two halves of the membranes.

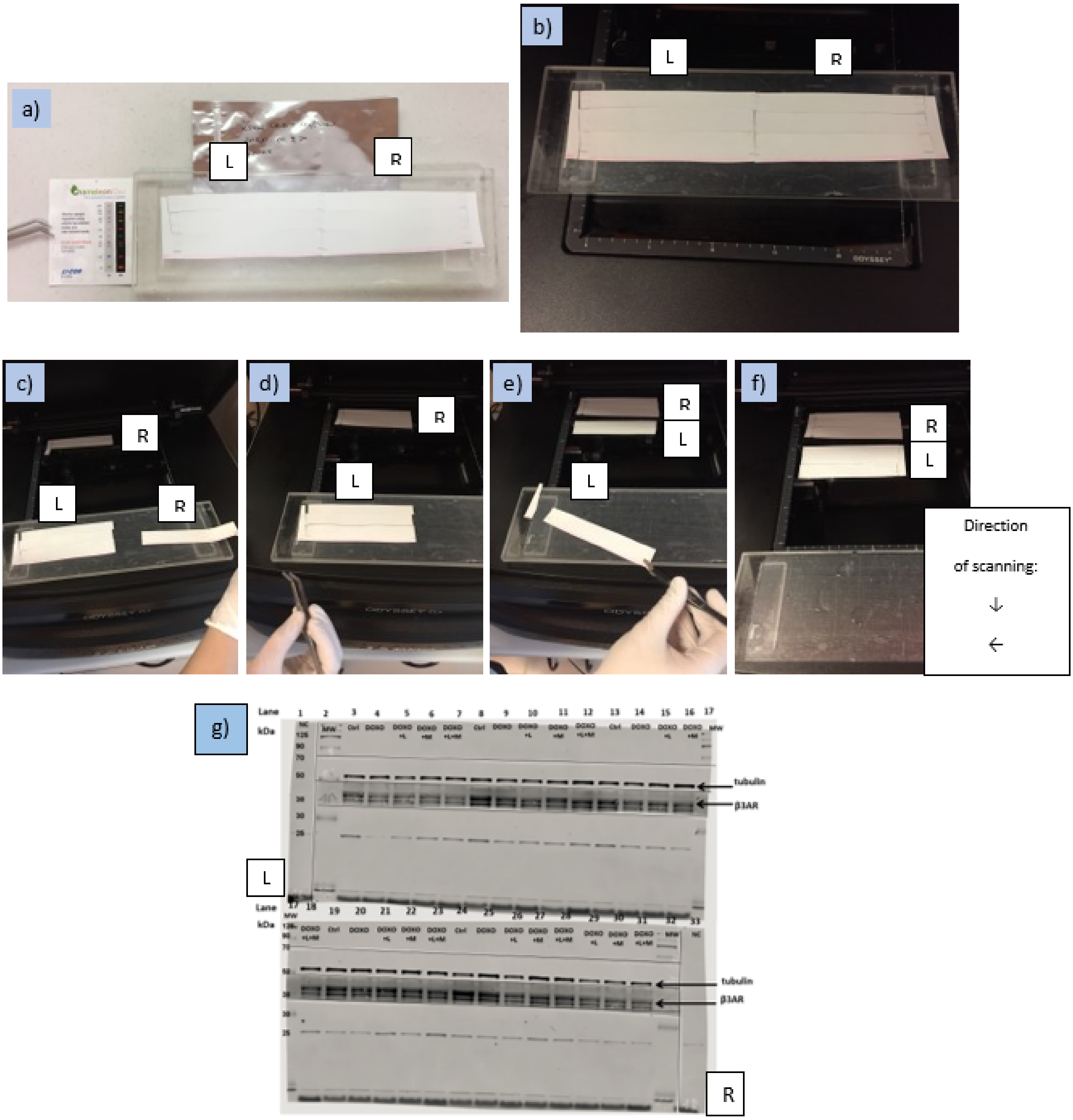

Figure S6. Examples for physical cutting (a-b) and positioning of the membranes into the Odyssey CLx machine (c-f). Scanned image of a membrane originating from the present project (g). L: left, R: right.

12. Western blot images provided in Supplementary Figures (Figure S1-S5) are the original uncropped and unmodified scans of the nitrocellulose membranes created during our present experiments. To help the understanding, we numbered the lanes continuously on the Ponceau-stained membranes and digital scans of the membranes and marked the experimental groups where each sample belonged and the place of the negative control (NC) or molecular weight marker (MW). We subtitled the protein of interest and loading background proteins on each gel. Different parts of the same membrane are divided by black lines. These scanned digital images were not cropped or digitally modified at all.
